# Seasonal migrations of the European sea bass (*Dicentrarchus labrax* L.) in UK and surrounding waters

**DOI:** 10.1186/s40462-024-00482-w

**Published:** 2024-06-11

**Authors:** Serena Wright, Christopher A. Griffiths, Victoria Bendall, David Righton, Kieran Hyder, Ewan Hunter

**Affiliations:** 1 Centre for Environment, Fisheries and Aquaculture Science (Cefas), Pakefield Road, Lowestoft, Suffolk NR33 0HT UK; 2 Department of Aquatic Resources, Institute of Marine Research, Swedish University of Agricultural Sciences, Turistgatan 5, 453 30 Lysekil, Sweden; 3 Defra, Seacole Building, 2 Marsham St, London, SW1P 4DF UK; 4 School of Environmental Sciences, University of East Anglia, Norwich Research Park, Norwich, Norfolk NR4 7TJ UK; 5 Fisheries & Aquatic Ecosystems Branch, Agri-Food & Biosciences Institute, Newforge Lane, Belfast, BT9 5PX UK

**Keywords:** Sea bass, Migration, Site fidelity, Spawning, Connectivity

## Abstract

The movements and behaviour of mature European sea bass (*Dicentrarchus labrax* L.) in UK waters have not been studied extensively since a series of mark-recapture experiments during the 1970s, 80s and 90s. To better understand the timing and extent of seasonal migrations, 171 mature sea bass > 42 cm were internally tagged with floated electronic tags programmed to record temperature and depth, and released in the English Channel, in the southern North Sea and in the Irish Sea. Among the 48 tags returned to date, sea bass were at liberty for 370 ± 337 days and were recovered 172 ± 200 km from their respective release locations. Most tags were recovered from beaches (54%), or via the fishery (44%). A comparison of the reconstructed tracks from returned electronic tags with the recapture locations of 237 mark-recapture returns (6.5%) from 3615 sea bass released between 1970 and 2020 showed strong overlap. Seasonal movements between shallow areas (Q2–Q3) and deeper spawning areas (Q4–Q1) were accompanied by elevated vertical swimming speeds and average water temperatures of 8.5 °C in the English Channel and Irish Sea, but lower temperatures in the North Sea. Movements between the Celtic Sea/Irish Sea and the North Sea and vice versa demonstrate high levels of connectivity in UK waters. We demonstrate that a proportion of sea bass remained resident within the North Sea throughout the year, with a strong suggestion that spawning might be occurring. These data have significant implications for the future sustainable management of sea bass stocks in UK and surrounding waters.

**Supplementary Information:**

The online version contains supplementary material available at 10.1186/s40462-024-00482-w.

## Introduction

Shifts in the spatial distributions of fish species in response to climate change are now widely recognised (e.g., [36, 39, 49, 56]). Altered distributions most frequently manifest in temperate latitudes (e.g. in the northeast Atlantic) as fish expand their population ranges northward due to widespread oceanic warming (e.g. [17, 52, 57]). Such range expansions can gradually alter community structure and composition, notably via interspecific interactions of predation [1, 18, 61, 68] and competition [4, 35].

The European sea bass (*Dicentrarchus labrax* L., hereafter ‘bass’) is a prominent example of a highly mobile northeast Atlantic range-expanding predator. In recent decades, bass have expanded northwards from a Mediterranean base, [69], supporting a recreational fishery since before the 1950s [73] and a rapidly growing UK commercial fishery from the early 1970s onwards [45], and more recently reaching the Norwegian fjords [30] and the Baltic Sea [3]. These movements have prompted several studies on bass movement and migration, with initial studies focussed on behaviour and distribution occurring in south western England [24], the southern and eastern coast of England [43] and on the western coasts of England and Wales [33]. These early mark-recapture experiments established a pattern of juvenile bass emigrating offshore from nursery areas after 4–5 years [43, 50] and adult bass migrating between coastal summer feeding areas and offshore winter/spring spawning and overwintering areas [44]. While providing a solid foundation for our understanding of bass behaviour and probable migration routes, these early experiments were unable to determine the full extent of feeding site and spawning area fidelity, the extent to which bass were seasonally resident in a given area, or to conclusively link observed migrations with reproductive activity. For example, it has long remained a point of conjecture as to the degree to which bass at the northern edge of their expanding distribution, notably the original southern North Sea colonists, remained there throughout the year to establish breeding populations, or retreated into warmer waters during winter [45].

The northern bass stock has regardless been heavily exploited, and is commonly targeted by both recreational and commercial fishers [12, 45]. Bass are highly prized by recreational and commercial fisheries [26, 55], resulting in high fishing pressure on the northern stock inhabiting the North Sea, English Channel, Celtic Sea and Irish Sea ([27]). A steep decline in stock size was observed from 2009–18 [28], which was thought to be driven by a combination of overfishing and a succession of poor year classes [27]. As a result, the European Union (EU) in 2015 introduced emergency management measures to reduce fishing mortality on bass for both commercial and recreational fisheries that included closed seasons, catch limits, bag limits and an increase to the minimum landing size that have been updated annually [29]. This reduced Sthe fishing mortality to acceptable levels, but year class strength has remained low meaning that their recovery is slow despite increases in stock size from 2019 onwards [29]. Despite the high level of interest in bass and their fisheries, significant gaps in our knowledge on basic life history characteristics, most notably concerning the movements, migrations and population dynamics of these higher latitude fish [6, 29]. Bass are a relatively slow-growing and slow-maturing species, and do not reach sexual maturity until between 4 and 8 years [51]. The stock in northern waters is also characterised by pronounced annual variation in recruitment, making it susceptible to overexploitation at low stock sizes [46].

Current levels of commercial interest, trends in stock status, and the emergency EU measures have all sparked a renaissance in research activity on this species. New research programmes have provided novel information on population structure ([29, 54]), localised residency and inter annual fidelity [8, 12, 15, 20], migration [12, 13] and seasonal behaviour (Herrah et al., 2017) in French waters, spawning [5, 22] and nursery areas [67], all of which add to our knowledge on the life history and behaviour of this species. Most recently, de Pontual et al. [13] published the results of a study in which 1220 mature bass were tagged with electronic data storage tags (referred to as “electronic tags” in this study) at ten locations along the breadth of the French coastline from Dunkirk in the north to Capbreton in the south. Results from this work provide clear evidence of bass as a partially migratory species, with migrants exhibiting strong fidelity to summer feeding and winter spawning areas [13].

Continuing this trend, we here present the results of a seven-year research programme during which 171 mature bass were tagged and released from the UK coast between 2014–2019, in the southern North Sea, the western English Channel, and the Irish Sea. Consequently, the results from this study closely complement the data from de Pontual et al. [13]. The aims of this study were to better understand the timing and extent of seasonal migrations by bass in UK waters, and to provide new insights into spatial stock structure and connectivity between different regions. Furthermore, whilst most of our results relate to novel observations from electronic tags, we also compared these new findings with the results from historical mark-recapture tag returns from bass released in the same geographical domain. The results of this study have important implications for the management of European bass in UK and surrounding waters and will further complement ongoing studies on bass movement and behaviour.

## Methods

### Fish capture and tagging

#### Tagging with electronic tags 2014–2019

**Fig. 1:**
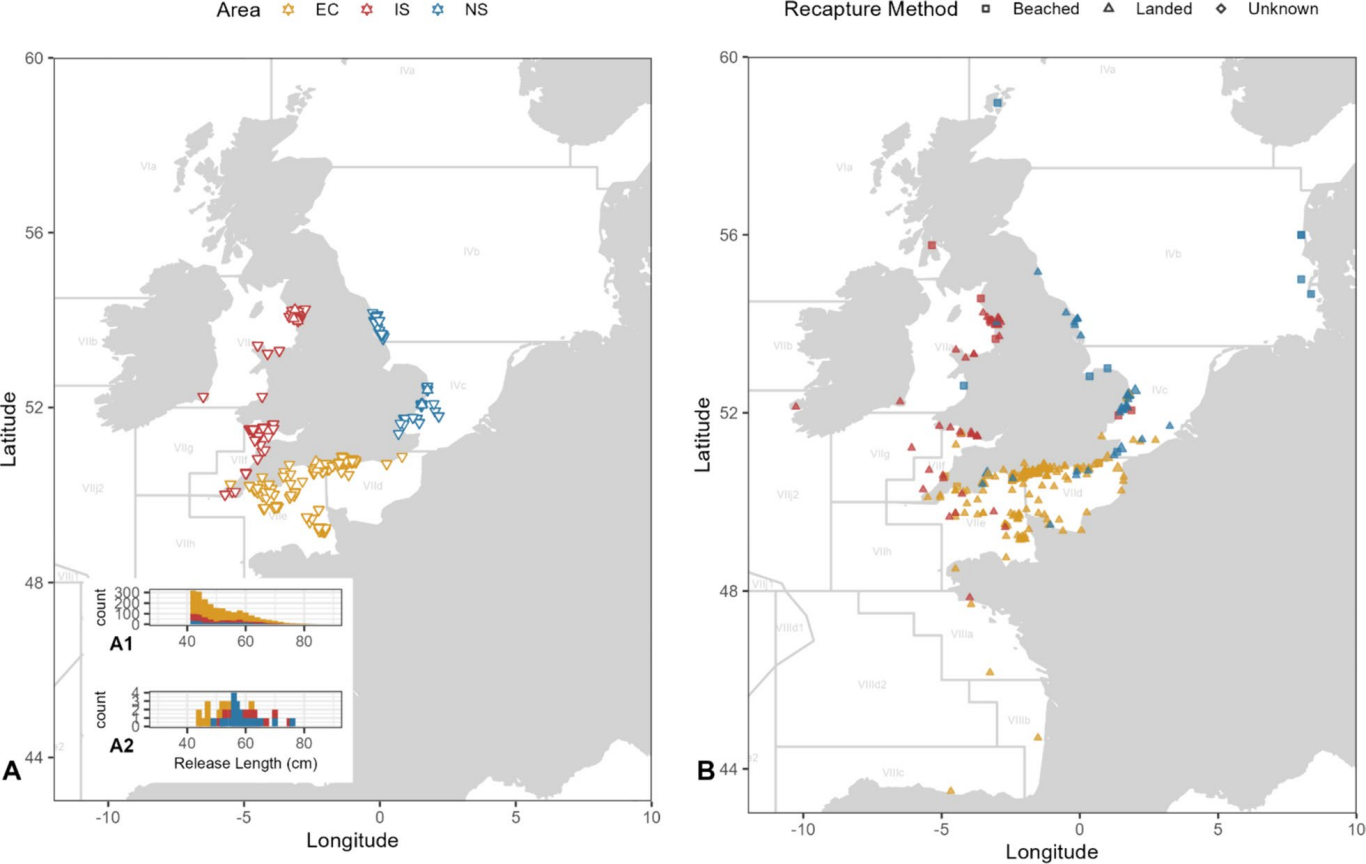
**A** Release locations of bass tagged with electronic ($\Delta$) and mark-recapture tagged ($\nabla$) bass in the North Sea (NS), English Channel (EC) and Irish Sea (IS). Size frequency of release length (TL) for mark-recapture tagged bass (A1) and electronically tagged bass (A2). **B** Recapture locations for electronically tagged and mark-recaptured bass released from the North Sea, Irish Sea and English Channel (colour denotes the release area for the individual). Recapture method is denoted as the symbol reflecting beached, landed or unknown recovery methods. ICES divisions are labelled in grey

For electronic tag deployments, mature bass were tagged and released from three areas around the English coastline between 2014 and 2019 (Fig. 1). Most individuals were captured for tagging by rod and line, but occasionally by net or longline (see Table 1 for details). In the English Channel (Weymouth; ICES division VIIe; 04/11/2014–27/11/2014), 48 bass (50 ± 5 cm Total Length; TL) were released. In the southern North Sea (Lowestoft and Orford; ICES division IVc; 13/05/2015–19/05/2017), 64 bass (59 ± 6 cm TL) were released. Finally, in the Irish Sea (Barrow; ICES division VIIa; 18/06/2017–18/07/2018) 59 bass (62 ± 7 cm TL) were released. All bass tagged were over 42 cm TL.

**Table 1:** Summary details of bass tagged with electronic tags and subsequently recovered, indicating the unique ID (ID), the date and location that the bass were released, total lengths, recovery methods (landed by net, rod and line, trawl, or found on a beach), whether the fish was predated, the distance from release location, time at liberty before tags were returned, the fate of the bass and the behavioural strategy (short distance migrators [Sho. Mig], intermediate migrators [Int. Mig] and long distance migrators [Lon. Mig])

ID	Release						Recovery method (and drift duration in days)	Predation (days in predators stomach)	Maximum depth (m)	Distance recovered (Km)	Time at liberty (days)	Fate	Max distance from release (km)	Strategy
	Location	Date	Latitude (N)	Longitude (E)	Length (cm)	Sex								
A10903	Eng. Cha	04/11/2014	50.621	− 2.274	52		Otter Trawl	F	76	41	300	Caught	138	Int. Mig
A10904	Eng. Cha	04/11/2014	50.621	− 2.274	44		Gill Nets	F	94	234	382	Caught	374	Lon. Mig (NS)
A10898	Eng. Cha	04/11/2014	50.621	− 2.274	52		Rod & line	F	79	4	698	Caught	268	Lon. Mig
A10913	Eng. Cha	04/11/2014	50.621	− 2.274	51		Rod & line	F	49	16	46	Caught	73	–
A10892	Eng. Cha	05/11/2014	50.688	− 2.217			Beached (202)	F	42	188	24	Prem	116	–
A10906	Eng. Cha	05/11/2014	50.688	− 2.217	53		Beached (35)	F	86	13	708	Prem	263	Lon. Mig
A10880	Eng. Cha	05/11/2014	50.688	− 2.217	55		Beached (135)	T (10.36)	83	206	292	Predated	109	Int. Mig
A10883	Eng. Cha	05/11/2014	50.688	− 2.217	47		Beached (701)	F	107	283	> 787	Unknown	419	Lon. Mig (CS)
A10893	Eng. Cha	05/11/2014	50.688	− 2.217	62		Long line	F	–	253	1097	Caught	–	Tag lost
A10889	Eng. Cha	05/11/2014	50.688	− 2.217	48		Rod & line	F	105	78	225	Caught	106	Int. Mig
A10881	Eng. Cha	26/11/2014	50.768	− 2.354	47	M	Beached (134)	F	103	155	678	Prem	122	Int. Mig
A10930	Eng. Cha	27/11/2014	50.591	− 2.318	44		Fish Market	F	57	–	34	Caught	45	–
A10943	Eng. Cha	27/11/2014	50.591	− 2.318	45		Beached (9)	F	78	214	284	Prem	379	Lon. Mig (NS)
A10950	Eng. Cha	27/11/2014	50.768	− 2.354	47	F	Beached (356)	–	–	159	1120	–	–	Error
A10952	North Sea	13/05/2015	52.06	1.55	76		Beached (58)	T (0.84)	57	537	397	Predated	411	Lon. Mig
A10920	North Sea	13/05/2015	52.06	1.55	63		Otter Trawl	F	59	12	364	Caught	45	Sho. Mig
A10932	North Sea	13/05/2015	52.06	1.55	58		Gill Nets	F	53	4	373	Caught	45	Sho. Mig
A10941	North Sea	13/05/2015	52.06	1.55	65		Beached (807)	T (1.82)	–	819	617	Predated	–	Error
A10978	North Sea	18/05/2017	52.4	1.75	52		Beached (12)	F	92	256	566	Caught and discard	152	Int. Mig (EC)
A10939	North Sea	24/05/2015	52.396	1.753	57		Fixed Nets	F	40	134	156	Caught	73	–
A10918	North Sea	24/05/2015	52.396	1.753	62	F	Beached (81)	F	95	84	748	Prem	450	Lon. Mig (EC)
A10947	North Sea	24/05/2015	52.396	1.753	52		Beached (2)	F	116	451	330	Prem	668	Lon. Mig
(EC & CS)														
A10931	North Sea	24/05/2015	52.396	1.753	58		Long line	F	57	2	368	Caught	41	Sho. Mig
A10979	North Sea	27/05/2015	52.396	1.753	70	F	Long line	F	56	31	218	Caught	21	Sho. Mig
A109742	North Sea	27/05/2015	52.396	1.753	56		London draw	F	–	–	58	Caught	–	–
A10978	North Sea	27/05/2015	52.396	1.753	59		Beached (12)	F	92	143	192		152	Int. Mig (EC)
A10961	North Sea	27/05/2015	52.396	1.753	49	M	Beached (120)	F	–	571	2	Death	–	–
A10964	North Sea	27/05/2015	52.396	1.753	56	M	Beached (128)	F	50	571	800	Prem	52	Int. Mig
A10989	North Sea	27/05/2015	52.396	1.753	55	M	Beached (72)	F	76	503	728	Prem	252	Lon. Mig (NS)
A10974	North Sea	27/05/2015	52.396	1.753	56		Beached (34)	F	–	106	68	Prem	–	–
A10958	North Sea	27/05/2015	52.396	1.753	57		Otter trawl	F	69	2	339	Caught	533	Lon. Mig (EC)
A10988	North Sea	27/05/2015	52.396	1.753	60		Rod & line	F	95	421	430	Caught	387	Lon. Mig
(EC & CS)														
A10991	North Sea	27/05/2015	52.396	1.753	61		Gill Nets	F	80	0	1435	Caught	214	Int. Mig (EC)
A12694	North Sea	18/05/2017	52.082	1.56	57		Unknown	F	–	55	32	Caught	–	–
A10963	North Sea	27/05/2015	52.396	1.753	56	F	Beached (5)	–	–	37	675	–	–	Error
A10955	North Sea	19/05/2017	52.07	1.513	53		Landed	F	–	–	55	Caught	–	–
A13607	Irish Sea	20/06/2017	53.991	− 3.025	70		Beached (15)	F	–	374	36	Death	–	Error
A13578	Irish Sea	20/06/2017	53.991	− 3.025	64		Beached (128)	T (1.59)	–	2	2	Predated	–	–
A13573	Irish Sea	20/06/2017	54.058	− 3.229	53		Gill Nets	F	14	16	57	Caught	31	–
A13592	Irish Sea	21/06/2017	53.991	− 3.025	55		Beached (75)	T (0.68)	–	37	67	Predated	–	–
A13602	Irish Sea	18/06/2017	54.02	− 3.02	61		Rod & line	F	141	8	415	Caught	419	Lon. Mig (CS)
A13601	Irish Sea	16/07/2018	54.237	− 3.127	62		Gill Nets	F	–	19	104	Caught	–	–
A13623	Irish Sea	21/06/2017	54.031	− 3.165	60		Rod & line	F	95	0	293	Caught	355	Lon. Mig (CS)
A13629	Irish Sea	18/06/2017	53.9833	− 3.0166	75		Beached (310)	–	115	248	> 630	Prem	350	Lon. Mig (CS)
A13624	Irish Sea	18/06/2017	53.9833	− 3.0166	59		Rod & line	F	125	0	736	Caught	346	Lon. Mig (CS)
A13618	Irish Sea	21/06/2017	54.0333	− 3.1666	51		Beached (10)	F	–	8	18	Death	–	–
A13589	Irish Sea	19/06/2017	53.991	− 3.025	67		Beached (169)	T	–	263	93	–	–	–
A15274	Irish Sea	18/07/2018	54.1166	− 3.0166	63		Beached (57)	F	103	62	275	Prem	283	Lon. Mig (CS)

After capture, all bass were brought slowly to the surface to avoid rupture of the swim bladder and those in good condition (alert with no significant injuries or bleeding) were placed into 1000 L holding tanks. Bass were then anaesthetised in a shallow (20 cm) bath containing 2-phenoxy-ethanol (0.5 ml^–1^). A small incision was made anterior to the anus on the midline of the ventral surface, and a Cefas buoyant G5 Data Storage Tag (DST) (Cefas Technology Ltd, Lowestoft UK) was then inserted into the intra-coelomic cavity [53]. Bass tagged after 2017 were also treated with local analgesia (lidocaine hydrochloride at a concentration of 1mg/ml and 1ml/kg). The wound was then sutured with polydioxanone, 4.0 metric, absorbable monofilament (Ethicon) sutures. Following tagging, the bass were placed in a recovery tank until they had regained equilibrium and were considered fit for release to the wild (up to 10 min, [53]). All tagging procedures were carried out by trained and competent scientists under Home Office project licence PPL 70/7734, with tagging methods similar to methods used in Quayle et al. [53] and [70].

The tags were programmed to record depth and temperature at 1- and 10-min intervals, respectively. Physical recovery of tags was necessary to retrieve the archived depth and temperature information. Tag-return was encouraged through a reward scheme advertised on posters distributed throughout UK port offices, the Marine Management Organisations (MMO) and Inshore Fisheries and Conservation Authorities (IFCAs). Rewards included the market value of the fish, €100 for the tag return and entry into a €1000 lottery.

#### Mark-recapture tagging data 1983–2020

For mark-recapture tagging, bass were tagged around England, Wales, the Channel Isles and southern Ireland between 1983 and 2020. Methods used for conventional tagging are summarised for releases between 1970 and 1984 in Pawson et al. [43], and between 2000 and 2005 in Pawson et al. [46]. In brief, bass were caught in a range of commercial (mainly trawls) and recreational fishing gears and were tagged with various mark-recapture tag types (e.g., abdominal anchor tag made by Hallprint PTY Ltd., Holden Hill, South Australia). Most tags were attached to the left flank of the fish, midway between the distal tip of the pelvic fin and the vent, the tags consisting of a coloured streamer with a unique serial number and recapture contact details, and an insert anchor. Those captured by trawl were held for at least 1 h in a tank of refreshed seawater prior to tagging to ensure that only fish most likely to survive were tagged. For the full details on the tagging process and licenses, we refer reader to Pawson et al. [43], [46] and Pickett et al. [50] and references listed therein. Releases between 2005 and 2020 follow the methods reported in Pawson et al. [46]. Only mark-recapture tagged bass with a TL > 42 cm were considered in the present study, to ensure consistency with the electronic tagged bass, with a total of 3,615 bass released in the English Channel (2,580), North Sea (322) and Irish Sea (713), as summarised in Table 2.

**Table 2:** Historical mark-recapture tag release and recapture information by release area between the Irish/Celtic Sea (ICS), English Channel (EC) and North Sea (NS) by ICES division

Release area	Release region	Number	ICS			EC			NS		BB		
			7.a	7.g	7.f	7.h	7.e	7.d	4.c	4.b	8.a	8.b	8.cC
ICS	7.a	400	10	1	1	0	3	0	0	0	0	0	0
	7.f	313	1	1	15	0	2	0	0	0	1	0	0
EC	7.e	2017	0	0	3	0	75	26	6	0	2	1	1
	7.d	563	0	0	0	0	8	62	0	0	0	0	0
NS	4.c	205	0	0	0	0	1	3	6	0	0	0	0
	4.b	117	1	0	0	0	1	1	3	8	0	0	0
	Total	3615	12	2	19	0	90	92	9	8	3	1	1

Data from [43, 46]

### Analytical methods

#### Bass fate

To classify the fate of released bass, two analysis steps were undertaken using returned tags based on (1) the tag recovery method and (2) the depth and temperature recorded by the tag. These two criteria were used to identify the point at which beached tags detached due to (A) fish death (due to predation), (B) commercial/recreational capture (with the tag removed and discarded), or (C) premature unspecified tag detachment:

A. Predation events by mammals were identified by assessing whether the depth and temperature changed from ‘typical’ bass behaviour to behaviours more typical of predators. For mammals this included vertical profiles showing frequent movements into surface waters and high temperatures (> 35 °C).
B. Capture followed by discarding was identified by looking at the temperature range on the day that tags started floating, which provides an indication of whether tags were exposed to the air (which results in a marked change in temperature, indicative of capture, Figure S1).
C. Premature detachments (resulting from death with no sign of predation) were explored further by comparing the behaviour of the bass to the behaviour of “typical” bass. “Typical” behaviours were identified using the daily proportion of time that individuals spent close to the assumed seabed (within 20% of the maximum depth), and within 5 m of the sea surface.

#### Horizontal movements—reconstruction of bass tracks from returned electronic tags

The migratory behaviour of each bass was reconstructed using a revised version of the Hidden Markov Model (HMM) described in Pedersen et al. [48] on a 5.2 km resolution grid. The HMM uses a novel Fokker–Planck based method incorporating previously described geolocation techniques [25, 41] and provides an estimate of an individual’s daily location based on its previous daily location and current behavioural state. The conditions experienced by the bass (depth and temperature) are used to inform a daily likelihood layer to improve the accuracy of location estimates. Behaviour within the model is defined by a diffusivity parameter, which governs the maximum distance that the modelled individual can move in any given day.

Updates to the model were the addition of sea-bed temperature and masking areas (see below). The underlying data layers used for the daily likelihood layer were bathymetry [21], tidal amplitude and phase (Oregon State University Tidal Prediction model; Egbert and Erofeeva [16]), sea surface temperature (Operational Sea Surface and Sea Ice Analysis database, Stark et al. [60]) and temperature at depth (Operational Mercator global Ocean analysis system, Lellouche et al. [37]). Matching depth, tide and temperatures were used to update daily likelihood layers:

1. Omission (masking) of regions shallower than the maximum recorded depth.
2. Higher likelihood (Gaussian distribution) given to regions with tidal signals identified as “waves” within the pressure data [25].
3. Higher likelihood (Gaussian distribution) given to regions with matching temperatures at corresponding depths.

Additionally, depending on the recapture source, i.e., whether the tag was recaptured by a fishing vessel (high confidence: < 5km error) or picked up from a beach (low confidence: > 200 km error) the distance from the recovery location was updated to reflect the level of confidence in the latitude and longitude on the day the tag was found. Once a likelihood layer was produced for all days at liberty (using steps 1 to 3 above), the behaviour of the bass was factored into the model (diffusivity parameter), reflected in the maximum travel distance permitted per day. Diffusivity was estimated using the method detailed in Pedersen et al. [48], such that two values were estimated corresponding to localised (resident) and migratory distances, respectively. Smaller values reflect restricted movement, with 0 being the same location as the previous day.

For each day, the HMM estimates a ‘most probable daily position’ including the error and uncertainty associated with the position estimate. The movement of an individual bass through time and space is also estimated by the HMM using the Viterbi algorithm, which reflects an individual's ‘most probable track’ and is not necessary a line drawn between the ‘most probably daily’ positions. Similar geolocation and reconstruction of track methods were used for electronically tagged yellowfin tuna (*Thunnus albacares*) in Wright et al. [71] and starry smooth-hound (*Mustelus asterias*) in Griffiths et al. [23]. Daily most probable positions were visualised using a 2D kernel density estimate with the kde2d function from the MASS package (version 7.3–58.1, 2022 [65]). One caveat of using a 2D kernel density estimate is that it assumes independence between daily positions. This assumption of independence is unlikely to be true in movement of individual bass and this needs to be acknowledged. Despite this, kernel density estimation remains a powerful tool to visualise space use, and is used with this motivation here.

To explore how the distance travelled by individual bass compared to the population overall, the maximum distance travelled by an individual was calculated as the straight-line distance between release location and the furthest estimated location. For every bass at liberty > 182 days (half a year), maximum distance was split into one of three categories reflecting short distance migrators (< 50 km), intermediate migrators (> 50 km and < 200 km) and long-distance migrators (> 200 km).

#### Identification of probable spawning locations

Previous studies have indicated that low water temperatures can affect gonad development [7], with bass maturation and spawning considered improbable at temperatures below 9 °C [14]. Between 1981–1984, English Channel bass spawning occurred at temperatures between 8.5 and 11 °C [62], with the distribution of eggs between February and June following the easterly incursion of the 9 °C isotherm (with maximum spawning activity in February). In the present study, the location of bass in Q1 (January to March) was used to identify potential spawning areas, and was compared to monthly depth, vertical speed and temperature experienced by individuals in different areas. Other behavioural metrics which may be indicative of spawning were extracted including vertical speed and maximum depth.

## Results

### Electronic tag recaptures, fish fate and vulnerability

Forty-eight electronic tags have been recovered (28% of the 171 released): 14 from the English Channel (14/48 = 29%), 22 from the North Sea (22/64 = 34%) and 12 from the Irish Sea (12/59 = 20%). Tag returns have yielded a total of 16,997 days of data (Table 1). Bass were at liberty between 2 and 1435 days (370 ± 337 days) and were recovered between 0 and 819 km (172 ± 200km) from their respective release locations.

The method of recovery for the electronic tags was from beaches (56%), or via the fishery (44%, which includes bass recovered from fish markets). By area of release, beach recoveries were highest in the Irish Sea (58%) compared to the English Channel (50%) and North Sea (55%). Tags recovered through the fishery were highest in the English Channel (50%) compared to the North Sea (36%) and the Irish Sea (42%).

Beach recovered tags were classified as caught and discarded (1/48 = 2%), predated (5/48 = 10%) or prematurely detached for unknown reasons which could include mortality or tag rejection (11/48 = 23%). By area of release, the proportion predated by region varied from 7% (1/14) in the English Channel to 9% (2/22) in the North Sea and 17% (2/12) in the Irish Sea. The depth and temperature time series for those predated bass are provided in Figure S2.

### Mark-recapture tag recaptures

To date, 245 mark-recapture tags have been recovered (7% of the 3,615 released): 109 from the English Channel (109/2580 = 4%), 23 from the North Sea (23/322 = 7%), and 31 from the Irish/Celtic Sea (31/713 = 4%). Bass were at liberty for up to 3,100 days (383 ± 419 days) and were recovered up to 520 km (74 ± 105 km) from their respective release locations.

Of the conventional tag returns, the numbers released and recovered by region are summarised in Table 2. All bass releases had returns from the same release area, so this section summarises where bass were recovered in other regions. Most bass were tagged in the English Channel (2580) followed by the Irish Sea (713) and the North Sea (322). English Channel (ICES divisions VIId and VIIe) released bass were recovered in the Irish/Celtic Sea (VIIf), North Sea (IVc), and the Bay of Biscay (VIIIa, VIIIb, and VIIIc). North Sea (IVb and IVc) released bass were recovered in the English Channel (VIId and VIIe), and the Irish/Celtic Sea (VIIa). Irish Sea released bass (VIIa & VIIf) were recovered in the English Channel (VIIe, VIIf) and the Bay of Biscay (VIIIa).

### Timing and extent of migrations

**Fig. 2:**
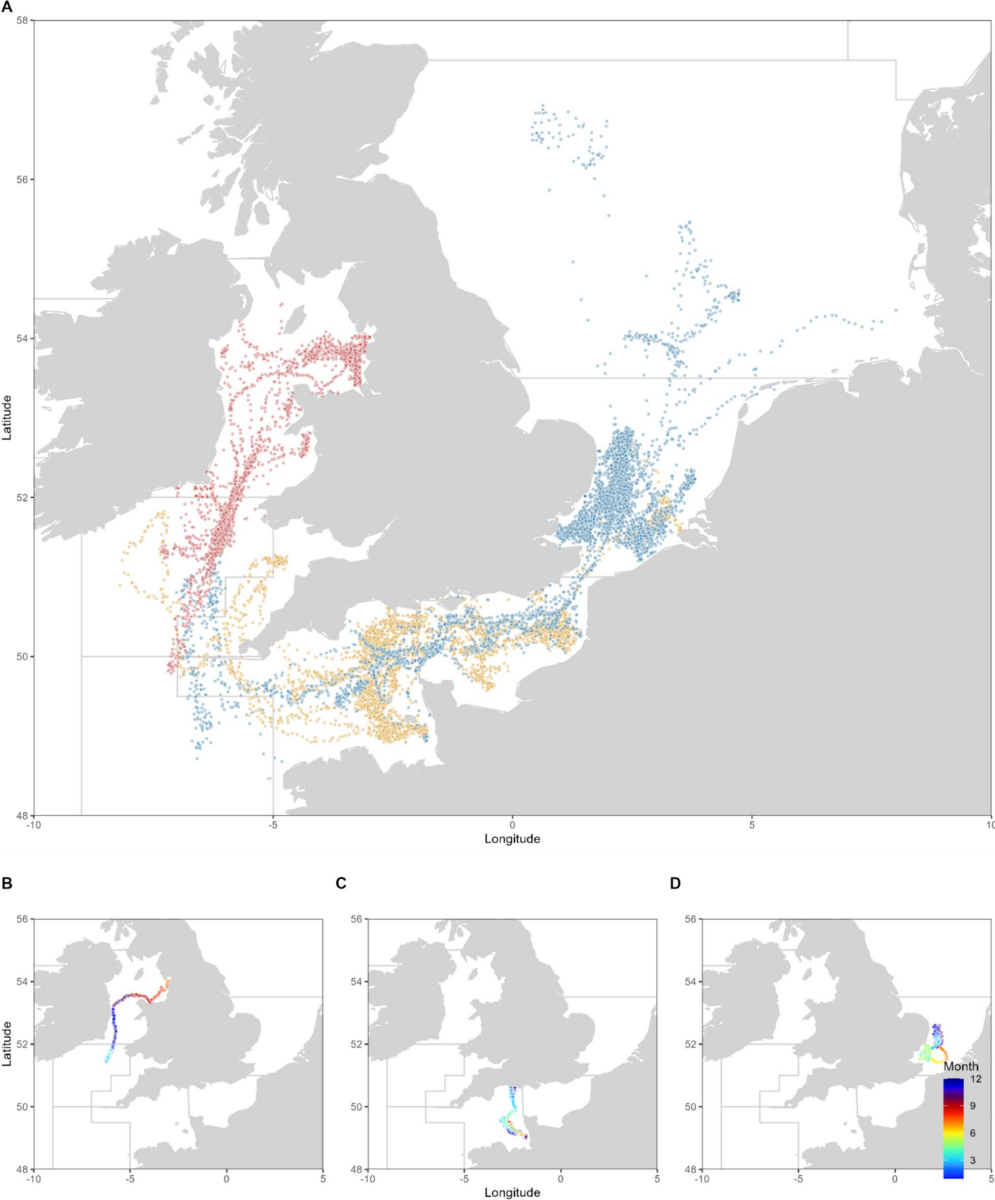
Daily most probable position estimates for **A** all bass tagged with electronic tags in the English Channel, VIIe (yellow), the Irish Sea, VIIa (green) and North Sea, IVc (blue). Examples of releases are provided in **B**–**D** with daily positions coloured by the month for bass 15,274 (**B**), bass 10,881 (**C**) and bass 10,932 (**D**)

Both recapture locations and reconstructed daily positions from electronic tags indicated a degree of mixing between areas (Figs. 1, 2, Table 1). The maximum straight-line distance travelled was 419 km in the English Channel, 668 km in the southern North Sea and 419 km in the Irish Sea (Fig. 1, Table 1). Results provide a strong indication of site-specific, seasonally directed movements that are indicative of feeding and spawning migrations.

**Fig. 3:**
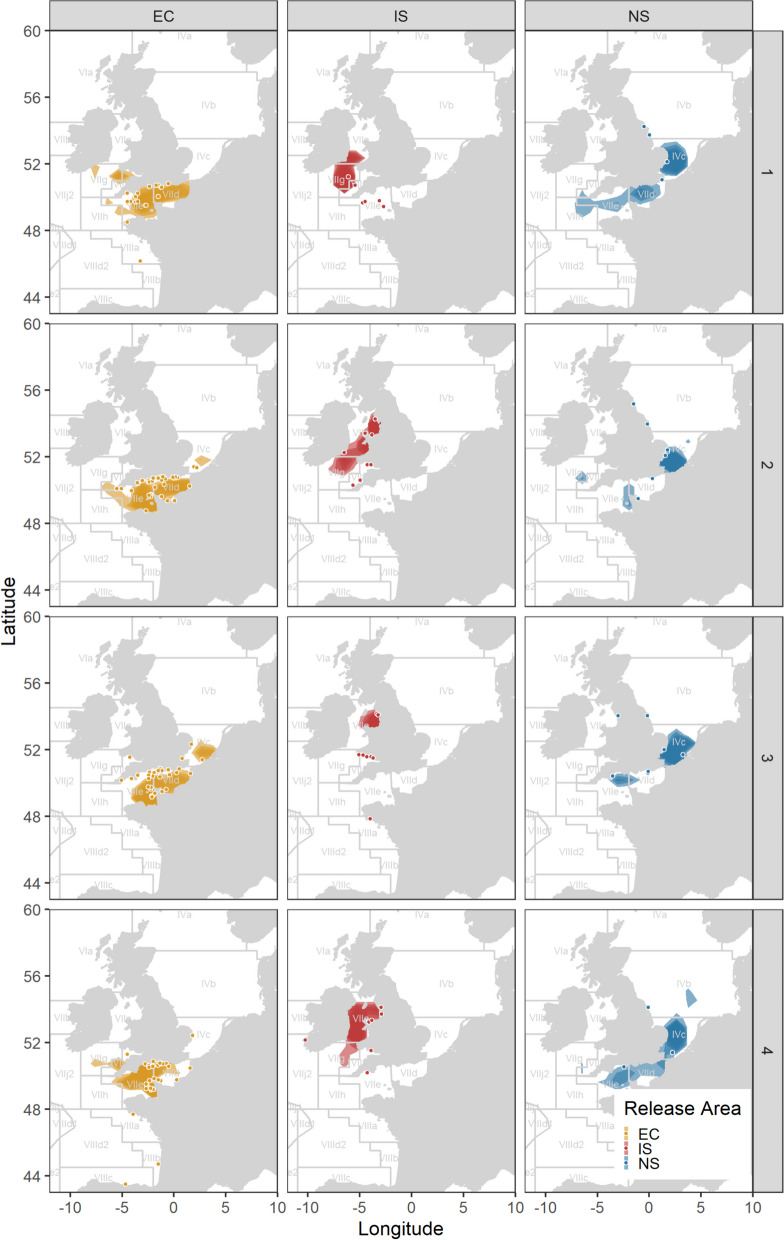
Kernel density of daily most probable position estimates of bass tagged with electronic tags by quarter and by release region (North Sea = NS, Irish Sea = IS, EC = English Channel). Points reflect the recapture locations of mark-recapture tagged bass

Of the electronic tags returned, nine English Channel released bass were at liberty > 6months. All English Channel released bass were considered intermediate to long distance migrators (n = 9), with migrations to the North Sea and the Celtic Sea (Table 1, Figs. 2, 3).

Fourteen North Sea bass were at liberty > 6months. Bass released in the North Sea were relatively evenly split between short distance migrators (29%, n = 4), intermediate migrators (28%, n = 4), and long-distance migrators (43%, n = 6). Migrations were observed into the central North Sea, the English Channel and Celtic Sea (Table 1, Fig. 2).

Four Irish Sea releases were at liberty > 6months. All bass released in the Irish Sea showed movements into the Celtic Sea (Table 1). Two bass (A13607 and A13589) were washed ashore in the southern North Sea (374–391 km from release), though both tags stopped recording prior to beaching. Migrations into the Irish Sea occurred in Q4, with bass remaining in the deep waters of the Celtic Sea until the end of Q1 before returning to the shallow waters of the Irish Sea (Fig. 2).

### Seasonal space use and hotspots

**Fig. 4:**
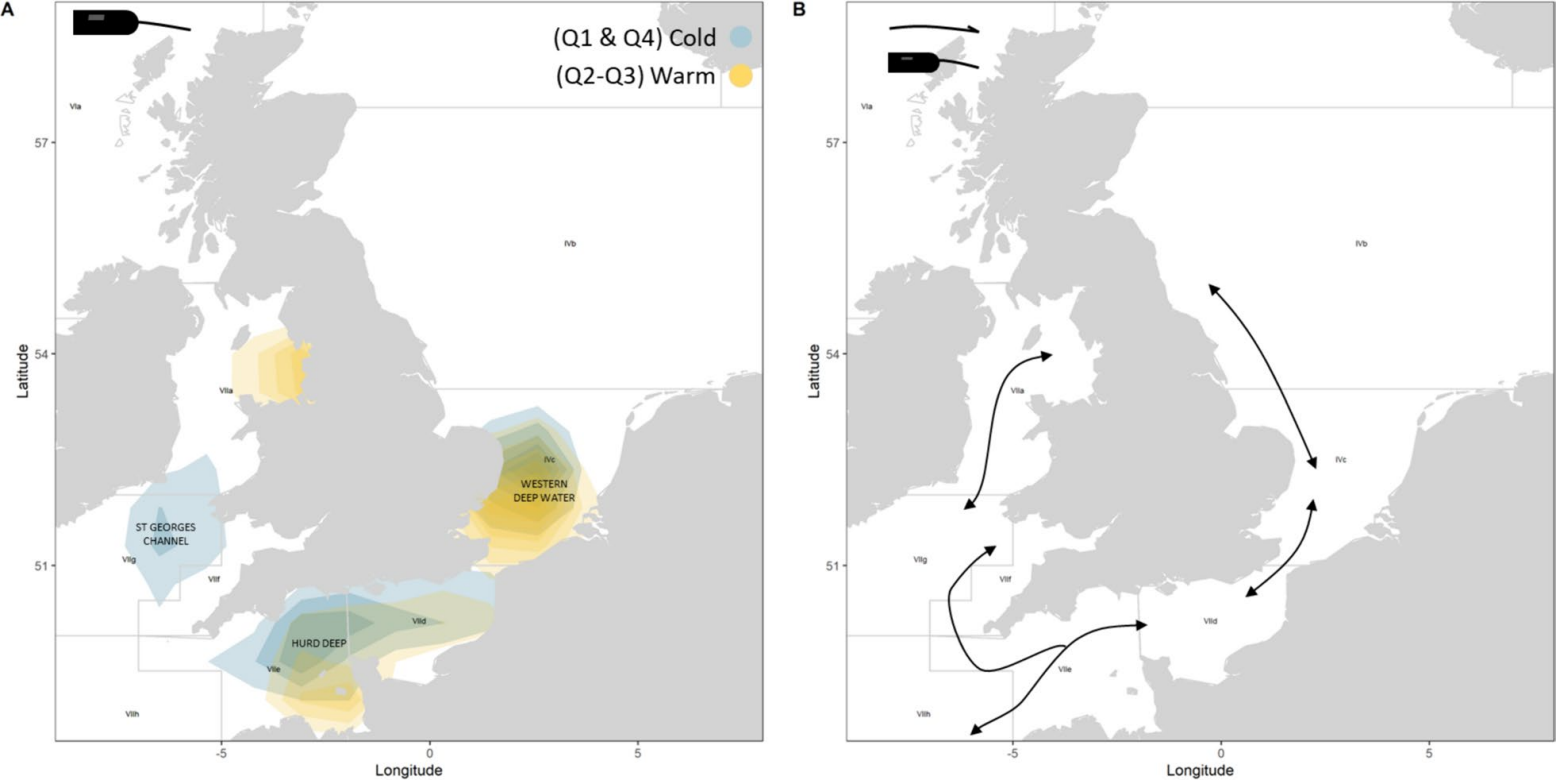
**A** Key areas by region indicating quarters 1 and 4 (cold) and quarters 2 and 3 (warm), and **B** directions of movement from key grounds for bass tagged with electronic tags. Q1 = January–March, Q2 = April–June, Q3 = July–September and Q4 = October–December

Data from both mark-recapture and from reconstructed movements from electronic tags were used to identify seasonal space use and hotspots (Figs. 3 and 4). Fish released in the English Channel were mostly recovered in the deep central English Channel (Hurd Deep) in Q1, where reconstructed fish tracks from the electronic tags also placed the majority of fish at this time. Quarters 2–4 had increased returns along the coastline, with higher dispersion to other regions (also observed from the mark-recapture data). Quarters 1 and 4 had conventional tag returns in the Bay of Biscay (n = 1 and 3, respectively).

Irish Sea released bass show coastal returns and association in Q2–Q4 with conventional tag data matching electronic tag daily positions and main hotspots in the Liverpool Bay area and along the western coast of the British Isles. That said, we did observe an increased range of coastal returns in the English Channel for conventional tags in Q4. In Q1, no returns were apparent from the Liverpool Bay area, but with mark-recapture tags recovered on the southwest coast and electronic tags showing a hotspot in the deep water of the Celtic Sea (St Georges Channel).

For North Sea bass, the spatial range of recoveries is mostly linked to the southern North Sea and English Channel for all quarters, with an increased range of returns for mark-recapture tagged fish throughout the year, including recoveries in the Liverpool Bay region in Q3 (n = 1). North Sea released bass seemed to aggregate in relatively deep waters of the Southern North Sea (Western Deepwater; Fig. 4A).

**Fig. 5:**
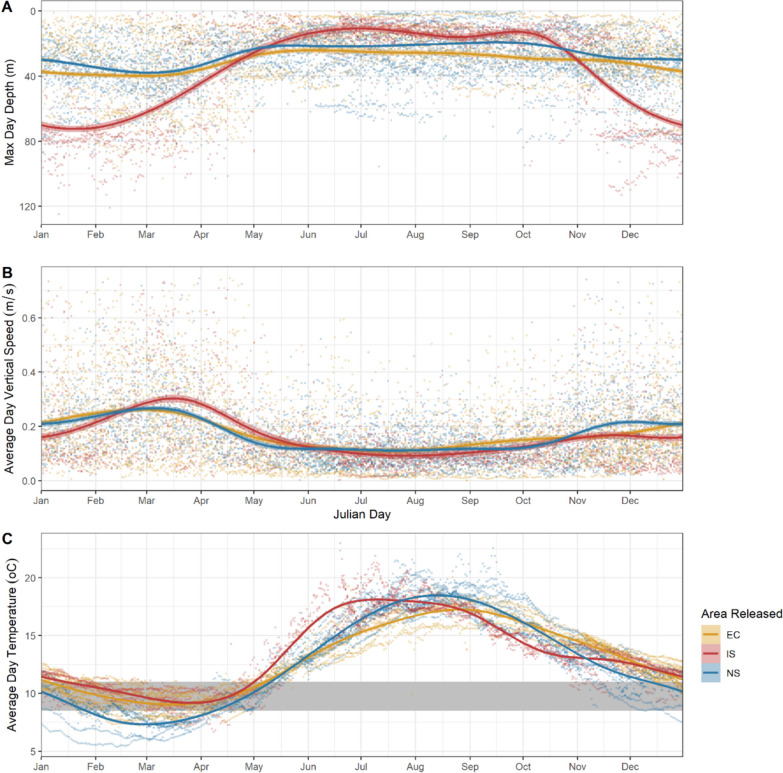
Daily maximum depth (**A**), average temperature (**B**) and average vertical speed (**C**) during the day for bass in the North Sea (NS), Irish Sea (IS) and English Channel (EC). A GAM smoother was applied to each group as shown by the solid line using a cyclic cubic regression spline. The filled area in temperature plot (B) represents temperatures between 8.5 and 11 °C

To help quantify the timing of movements to deeper offshore water, Fig. 5 and Table S1 provide the maximum depth, average vertical speed and average daily temperature for all bass, with individual maximum depths provided in Table 1. Representative traces of depth and temperature experienced are also provided in Figure S3. The maximum depth for bass that remained in the English Channel was 107m, for the Irish Sea was 141m and for the North Sea was 76m. Bass released in the North Sea and Irish Sea spent June to October in relatively shallow waters with more time spent at deeper depths between December and May (Fig. 5, Table S1). The time spent in relatively deep water in the English Channel was shorter than that for the other two regions, with a peak between February and March when the average depth exceeded 35m, compared to November to April in the Irish Sea, and December to March in the North Sea (Table S1). Vertical speeds show similar patterns to maximum depth, but with a more pronounced increase in average vertical speed for a short period between February and March for all bass groups (Fig. 5B). During this period, average vertical speeds exceeded 0.25m s^−1^ (Table S1).

Average daily temperature experienced by bass is shown in Fig. 5C. Average water temperatures remained about 8.5 °C for bass that were in the English Channel and Irish Sea. In the North Sea average temperature experienced went below 8.5 °C in February and March (Fig. 5C, Table S1), with March temperatures averaging 7.4 °C (Fig. 5C, Table S1).

## Discussion

Here we present results from the first extensive study of the movements and behaviour of mature bass in exclusively UK waters since [19] and Pawson et al. [46] released thousands of mark-recapture tagged bass during the 1970s, 80s and 90s. This provided the opportunity to re-examine the results of the historical mark-recapture data and observe the extent to which the original observations on bass migrations mapped onto the much more detailed records of individual activity gained from electronic tags. Our results demonstrate variability in the extent of migration at each of our release sites, between short and long-distance migrators. In agreement with past work, we observed seasonal movements, site fidelity, and a high level of connectivity in UK waters, with bass moving between the Celtic Sea/Irish Sea and the North Sea and vice versa. Moreover, for the first time, we find evidence that a proportion of North Sea fish remained resident within the North Sea throughout the year, suggesting this area may have become suitable for spawning. The data presented have significant implications for the future sustainable management of bass stocks in UK and surrounding waters.

### Seasonal movements and stock mixing

Seasonal movement between inshore shallow grounds in the summer to deeper grounds in the winter were evident in the horizontal movements of tagged bass. Bass demonstrated strong seasonality in behaviours, with short distance migrations (within 50 km of the release location) to migrations over hundreds of kilometres. The maximum straight-line distances travelled from the point of release were 419 km in the English Channel, 668 km in the southern North Sea and 419 km in the Irish Sea (Table 1). Therefore, distances are similar to those reported for bass released in other regions of the UK and north-west France, at about 600 km [12, 13, 53].

Previous studies indicate that the southwest (English Channel) and west coasts of the UK have high levels of immigration from other regions, in contrast to the southern North Sea, which has been described as a net exporter of bass [50]. Our data align with these previous findings, with the majority (67%) of the English Channel releases having remained resident within the English Channel throughout their time at liberty. In contrast, 54% of our southern North Sea releases involved migrations into the central North Sea (7%) and English Channel (47%).

A clear difference between the results of this study and the recently published results of an extensive study of the movements of bass in French waters [13] is that none of the electronically tagged bass in the UK study migrated into Bay of Biscay waters. This may have been due to differences in the timing of release (the French bass were released in June), the scale of release (a total of 1220 electronically tagged bass were released in the French study, 150 of which were released in the southern North Sea), or the location (the French bass were released off Dunkirk, on the cusp of where the North Sea meets the English Channel). However, given the scale of the French tagging study, which covered 10 release locations spanning northeast to southwest France [13], it is further notable that none of the reconstructed French bass tracks moved into St. Georges Channel and the northern Celtic Sea.

### Feeding and spawning grounds

Our data confirm previous observations of site fidelity to presumed summer feeding grounds as observed off the West Coast of France [12], southern Ireland [15] and in UK waters [13, 47]. Over the past few decades several sites have reported increased bass abundance during these summer feeding periods, including in the Dutch Wadden Sea [9], the Dutch Coast and in the Western Scheldt estuary [66]. The apparently coordinated arrival of bass from both North Sea and English Channel releases off the Western Scheldt in the early summer (late June/early July) further indicates that this region is important for bass populations. It is suggested that the timing of arrival corresponds well with the peak of an important brown shrimp fishery in this region, with crustaceans known to be an important food source for bass [38, 59].

Previous studies have also noted site fidelity to winter spawning grounds [12] with an indication that populations are spatially structured, which has implications for current stock delineation [13]. Bass have a latitudinal gradient in the onset of spawning, with spawning occurring earlier at lower latitudes [66]. In Ireland spawning occurs from April to mid-June [34], in the Bristol Channel between March and April [31], in Brittany and the English Channel, between February and May [10, 62] and in Cadiz between October and January [2]. Finally, surveys in the North Sea have found sea bass eggs and larvae in April and May [63, 64]. The onset of spawning is thought to be triggered by photoperiod, for example, at lower latitudes (e.g., the waters off Northern Portugal) where water temperatures are higher and are unlikely to restrict spawning behaviour, day length may be the most powerful determinant of the spawning season [66].

All fish released in the present study were above size at which 50% of the population are considered to be mature (> 40.65 scm; Table 1) [28], with behaviour indicative of breeding partial migration [58] where residents and migrants separate to breed. Bass were observed in deep offshore waters between December and May (North Sea and Irish Sea releases) and between February and May for English Channel releases. Therefore, bass tagged at higher latitudes (English Channel and Irish Sea) appear to start moving towards deeper spawning areas earlier in the year than their more southerly counterparts, a finding apparently at odds with previous studies [66]. The locations during the potential spawning period included the Hurd deep in the English Channel, St Georges Channel in the Celtic Sea and Western Deep Water in the southern North Sea (Fig. 3). During these periods when occupying deeper offshore habitats, bass occurred at depths down to 141 m, shallower than concurrent depths reported for bass from the West Coast of France [12], where some bass were found at depths more than 229 m. The shallower depths reported in this study likely reflects differences in accessible habitats for these individuals (depths in the southern North Sea for example rarely exceed 50 m). Noting that recent studies also highlight the English Channel as an important spawning area for bass Dambrine et al. [11].

The timing of movements to deep waters off the south coast of the UK correspond with the spawning grounds identified in Pickett et al. [50] and in previous studies of bass egg distribution [31]. The aggregation of bass in the Western Deep Water and St. Georges Channel during the spawning phase may indicate that these areas are important for bass from the southern North Sea and the Irish Sea respectively, with potential mixing of bass from all sites in the deep waters of the Celtic Sea region (St Georges Channel). In addition to day length, temperature has been hypothesised to drive spawning behaviour of bass, with spawning in the English Channel being restricted to temperatures between 8.5 and 11 °C [62], and with a progression of spawning distributions between February and June following the easterly incursion of the 9 °C isotherm [43]. In recent decades temperatures in the English Channel have significantly increased [40], and bass in the present study are shown to remain in water above the critical 9 °C threshold, aligning with results from Pawson et al. [43]. In contrast, bass that remained in the North Sea experienced temperatures below 9 °C. Further research should be undertaken to confirm whether bass are spawning in these areas at lower temperatures than previously thought. As further evidence that movement into deeper waters was associated with spawning activity, during the presumed spawning period the tagged bass exhibited increased average vertical swimming speeds, including those in the southern North Sea. The average vertical speeds exceeded 0.25 m s^−1^ between February and March for all bass groups (Fig. 5B). Bass may have increased vertical speeds during this time as they make vertical migrations up into the water column to breed. Accelerated vertical swimming has been linked with possible spawning events in a variety of other marine fish species including flatfish [72]. Moreover, in the work of Heerah et al. [32] on sea bass, changes in vertical activity and depth use are shown to be strongly linked to seasonal changes in functional behaviour (e.g., feeding, migrating and spawning) across individuals.

As the two tagging data sets were not directly comparable, the aim of this study was not to directly look for evidence of behavioural change over a period of approximately fifty years, since the first release of mark-recapture tagged fish [43]. Instead, we restricted our observations to examining the extent to which the distributions of all mark-recapture tag returns overlapped with the reconstructed tracks from electronic tag returns in space and time. Ultimately the two datasets comfortably coincided, and although as noted above, none of the bass tagged with electronic tags migrated into the Bay of Biscay, for example, this most likely reflects the huge disparity in the period of time over which mark recapture tags were released, and the relatively small number of fish tagged with electronic tags compared with thousands of mark-recapture tagged fish.

### Tag and data recovery

By October 2022, forty-eight of the deployed electronic tags had been returned (28% of the 171 released). Previous mark-recapture studies of bass report return rates of 12% from conventionally tagged bass around the UK [46]. Similarly electronic tags (without floats) deployed on cod in waters around the UK had have return rates of 15% [42]. The increase in the recovery rate of bass in the present study compared to historical mark-recapture studies likely relates to a number of factors including the use of floats to aid in fisheries-independent returns of tags. A recent study which tagged 1220 bass using the same floated electronic tags used in this study [13], released at 10 locations spanning the length of the French coastline, achieved an overall return rate of nearly 40%. The majority of tags in this study were recovered from beaches (54%), with the highest incidence of beach returns from tags deployed from the Irish Sea (58%) and lowest from the English Channel (50%). The high return rate from beaches shows that utilising tags with floats can significantly increase the return rates of electronic tags for species inhabiting similar areas, as shown in the studies by de Pontual et al. [12] where 36% of recoveries were attributed to the use of floats. High return rates are also likely linked to rewards, surface currents and the well-used beaches and coastal areas around the coast of the UK and wider Europe.

## Conclusions

This study highlights the connectivity of bass in UK waters and provides evidence of potential spawning regions in the Hurd Deep in the English Channel, Western Deep Water in the southern North Sea and St George’s Channel in the Celtic Sea. The Hurd Deep in the English Channel aligns with previous studies on spawning periods and areas defined in Pawson et al. [46]. The latter two spawning areas in the southern North Sea and Celtic Sea have not previously been identified as sites important to bass and warrants further investigation based on the limited number of tag returns to date. To help validate spawning behaviour and to quantify mixing rates between different areas, future work should be undertaken to combine these bass time series with other tagging programs covering similar areas in the Northwest Atlantic. Furthermore, high return rates from beaches indicate that studies using archival tags around the UK should consider using floats to help maximise return rates.

### Supplementary Information

**Additional file 1**.

## Data Availability

Data can be provided upon request with an overview of available datasets provided on the Cefas data portal.
