# Supplementary material for: Seasonal migrations of the European sea bass (Dicentrarchus labrax L.) in UK and surrounding waters
Source: Mov Ecol. 2024 Jun 11;12:45. doi: 10.1186/s40462-024-00482-w (PMC11167799; doi:10.1186/s40462-024-00482-w)
Supplement: Supplementary file 1 — Additional file 1. [file 40462_2024_482_MOESM1_ESM.docx]

Supplementary

Table S1. Daily maximum depth, vertical speed and temperature during the day for bass in the North Sea (NS), Irish Sea (IS) and English Channel (EC)

|  |  | Jan | Feb | Mar | Apr | May | Jun | Jul | Aug | Sep | Oct | Nov | Dec |
| --- | --- | --- | --- | --- | --- | --- | --- | --- | --- | --- | --- | --- | --- |
| Max depth (m) | NS | 35.91 | 41.08 | 38.58 | 32.77 | 22.00 | 24.07 | 25.89 | 24.92 | 27.76 | 30.89 | 29.24 | 36.44 |
|  | IS | 68.10 | 63.11 | 50.52 | 35.02 | 14.53 | 12.14 | 11.89 | 13.92 | 15.71 | 17.95 | 45.17 | 63.30 |
|  | EC | 30.77 | 36.28 | 38.66 | 24.95 | 23.32 | 21.01 | 20.90 | 20.41 | 18.65 | 22.15 | 27.51 | 30.89 |
| Average vertical speed (m s^-1^) | NS | 0.23 | 0.28 | 0.27 | 0.21 | 0.12 | 0.13 | 0.11 | 0.12 | 0.15 | 0.15 | 0.17 | 0.19 |
|  | IS | 0.20 | 0.26 | 0.31 | 0.24 | 0.13 | 0.11 | 0.09 | 0.10 | 0.11 | 0.13 | 0.18 | 0.15 |
|  | EC | 0.21 | 0.26 | 0.28 | 0.16 | 0.14 | 0.11 | 0.11 | 0.12 | 0.11 | 0.14 | 0.21 | 0.22 |
| Average Temperature (°C) | NS | 10.55 | 9.48 | 9.10 | 9.67 | 11.80 | 14.20 | 16.02 | 17.10 | 16.86 | 15.24 | 13.78 | 11.96 |
|  | IS | 11.01 | 10.21 | 9.39 | 9.67 | 13.39 | 17.34 | 18.19 | 17.64 | 15.65 | 13.60 | 12.93 | 12.02 |
|  | EC | 8.98 | 7.80 | 7.39 | 9.10 | 11.92 | 14.78 | 17.63 | 18.38 | 17.31 | 14.67 | 12.44 | 10.94 |


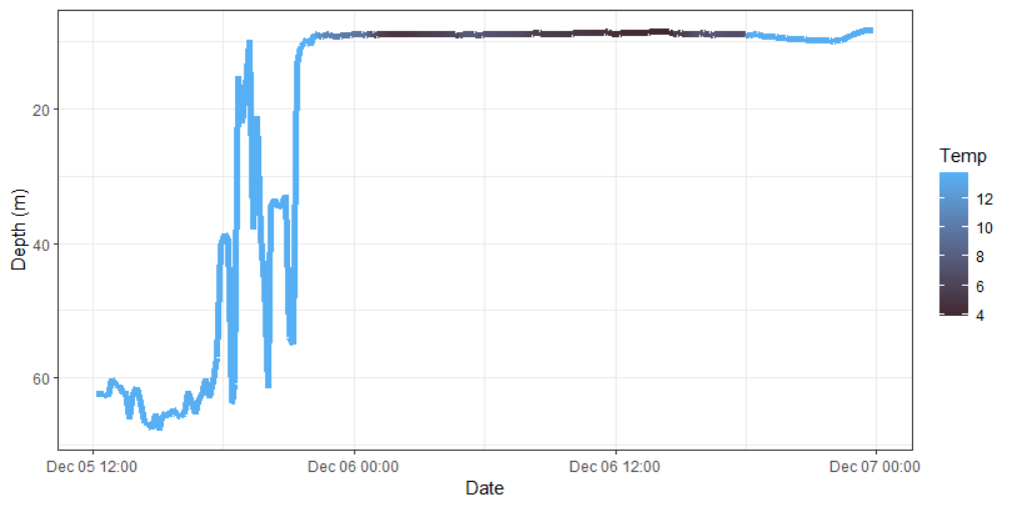


Figure S1. Fish caught and discarded (Tag 10978). Normal bass behaviour shown until 05/12/2015 at around 20:00. The depth trace and temperature indicate the bass in then put on ice until 06/12/2015 at around 18:30 when the tag is discarded into the sea before being washed up.


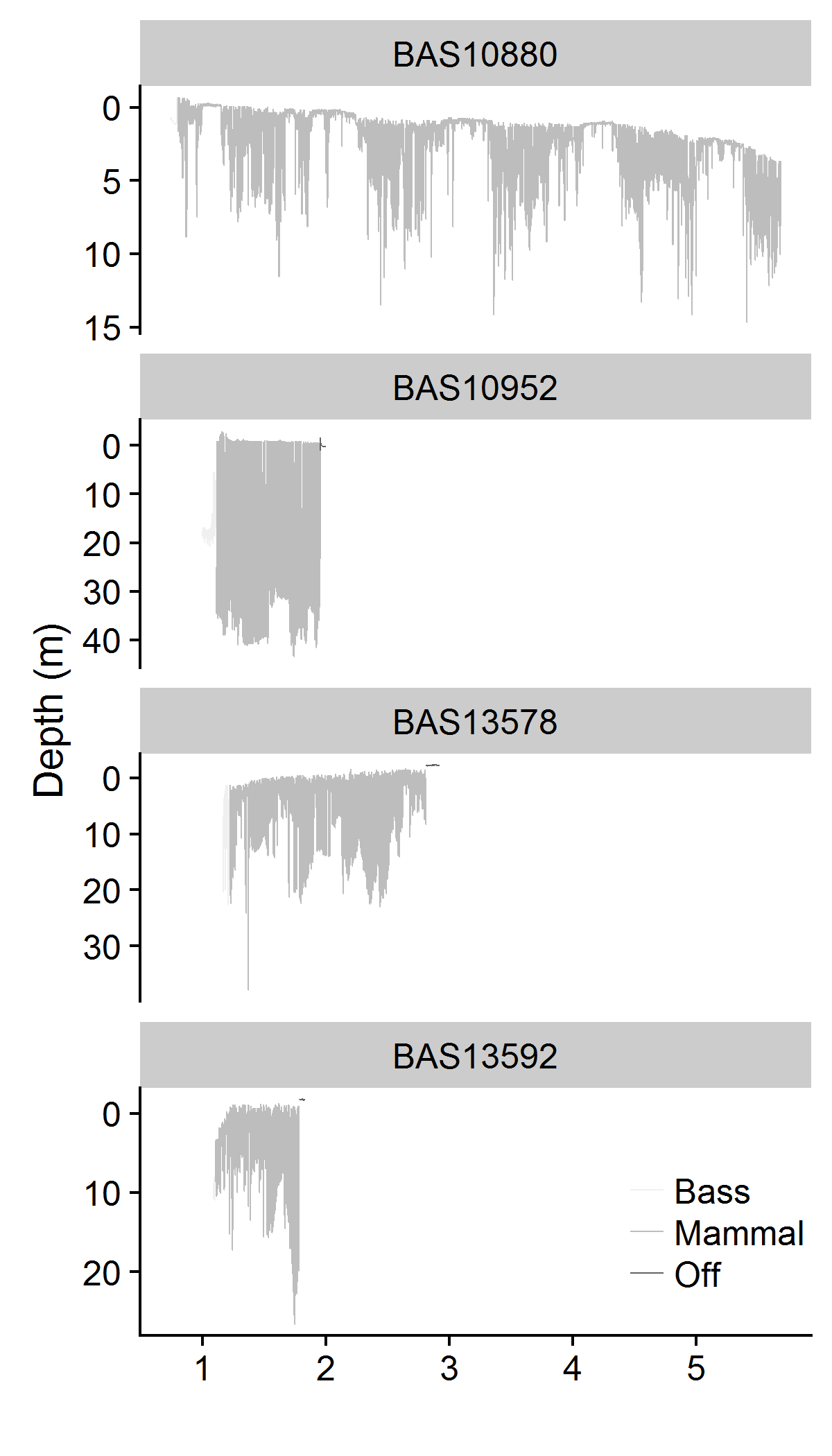

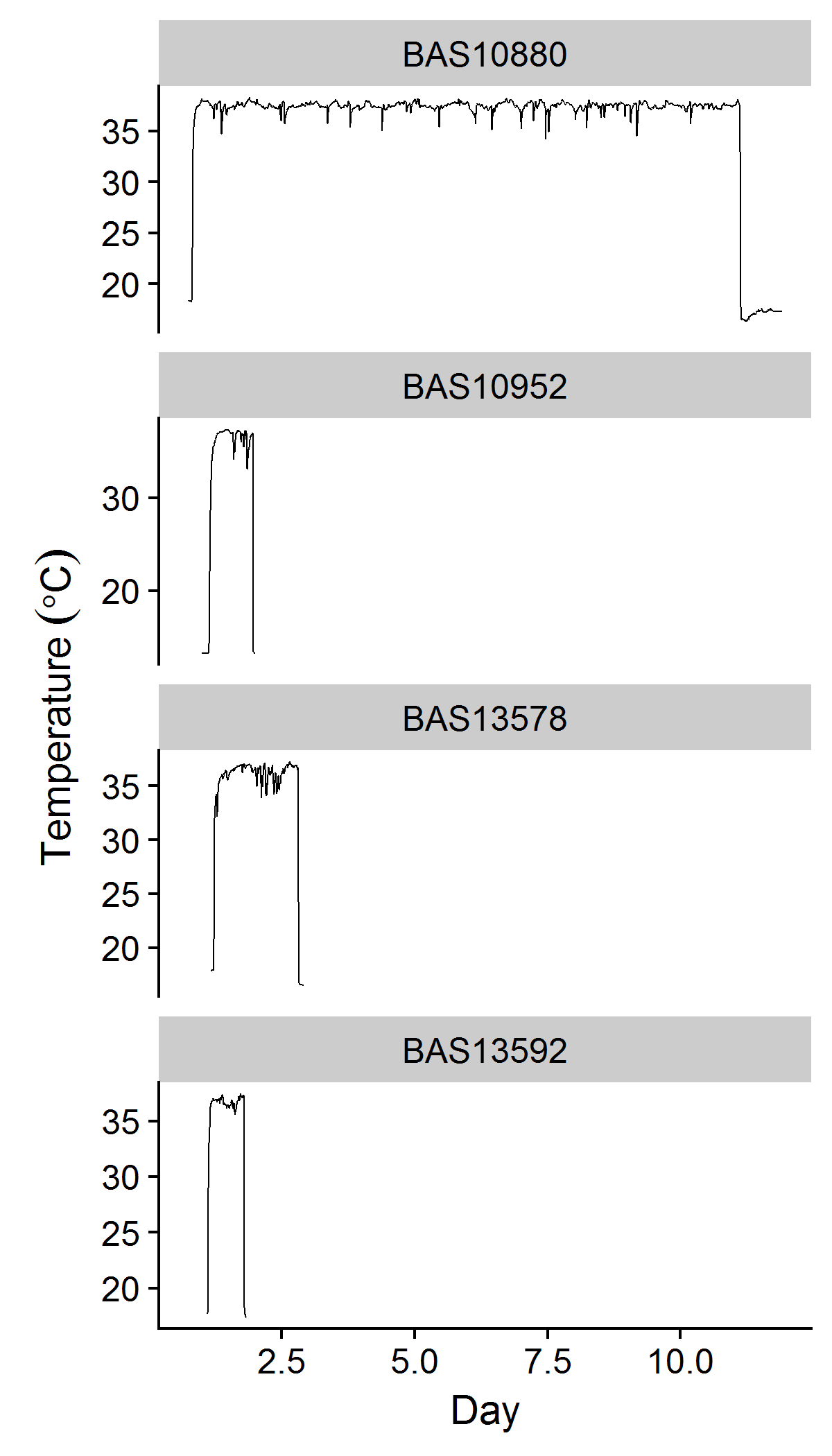


Figure S2. Predation events by marine mammals indicated by changes in depth and temperature.


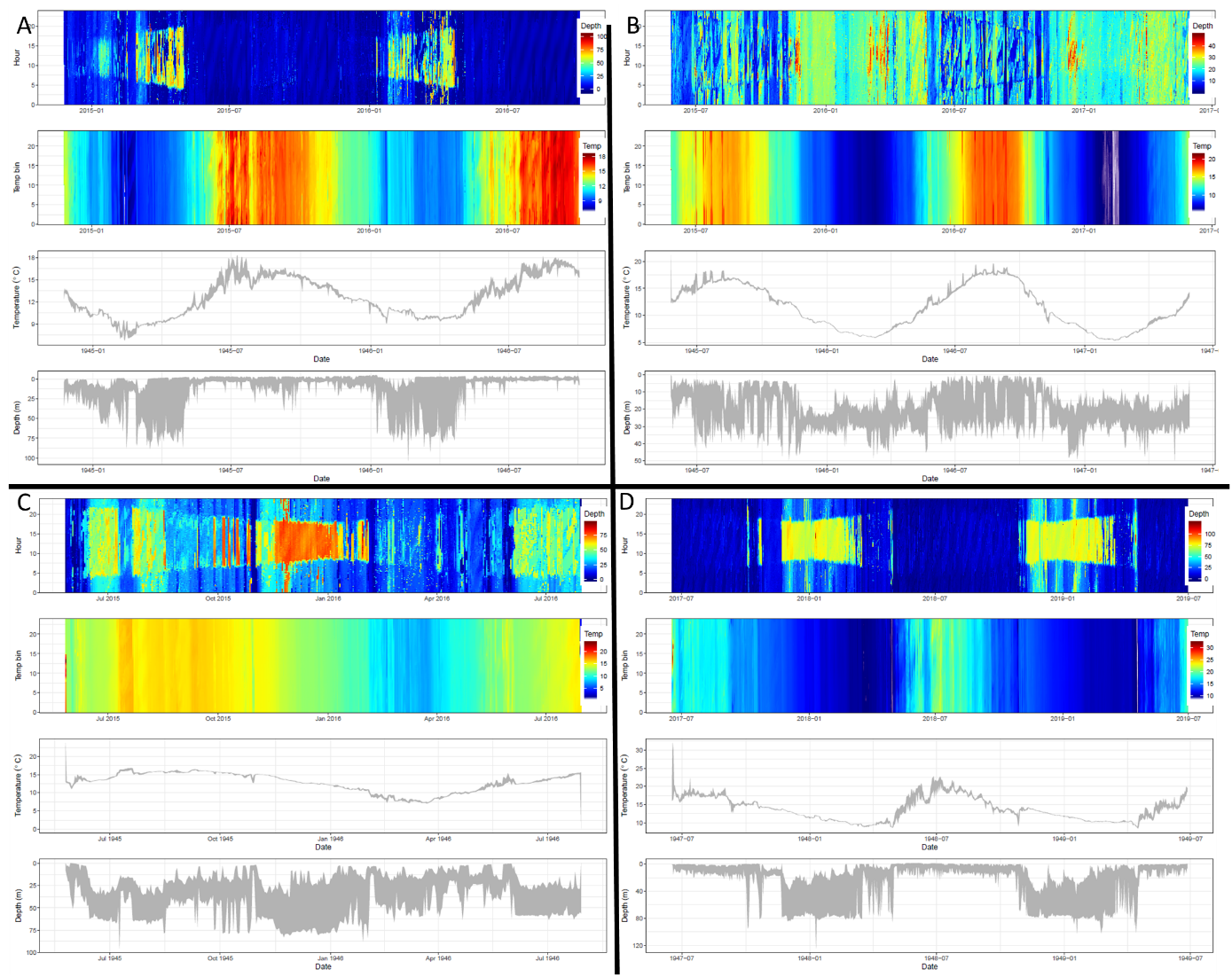


Figure S3. Summaries of bass behaviour for representatives released from the English Channel (A: Bass 10881), the Southern North Sea (B: Bass 10964 and C: Bass 10964) and the Irish Sea (D: Bass 13624). Summaries include depth and temperature actograms and the minimum and maximum temperature and depth experience of the bass by day.
